# Effectiveness of a Novel Multimodal Intervention for Family Caregivers of Persons With Age-Related Macular Degeneration: A Randomized Controlled Trial

**DOI:** 10.7759/cureus.72523

**Published:** 2024-10-28

**Authors:** Richard Kha, Ivan Jin, Diana Tang, Gerald Liew, Ashley Craig, George Burlutsky, Paul Mitchell, Bamini Gopinath

**Affiliations:** 1 Centre for Vision Research, Westmead Institute for Medical Research, Westmead, AUS; 2 Macquarie University Hearing, Macquarie University, North Ryde, AUS

**Keywords:** age-related macular degeneration (amd), cognitive behavioural therapy, family caregiver, guidance and counselling, quality of life (qol)

## Abstract

Purpose

Age-related macular degeneration (AMD) is a leading cause of visual impairment in older adults. Individuals affected by AMD often require regular physical and emotional support from family caregivers. Carers of people with AMD endure significant physical burdens, emotional distress, increased financial stress, and disruptions due to their lifestyle and retirement plans as a direct consequence of the AMD caregiving experience. Despite this, there are currently no interventions targeted toward family caregivers of AMD patients. We evaluated the efficacy of a novel intervention aiming to improve the burden and well-being of family carers of persons with AMD.

Methods

Family carers of relatives with AMD were primarily recruited through private eye clinics and randomized 1:1 to either receive a 10-week intervention of mail-delivered cognitive behavioral therapy (M-CBT) and optional telephone-delivered group counseling (n = 47) or to a wait-list control group (n = 47). Outcome measures were assessed pre-intervention (baseline) and six months post-intervention. These included treatment acceptability, caregiver burden, presence of depressive symptoms, self-efficacy, quality of life (QoL), and fatigue.

Results

A total of 94 participants were enrolled, with 47 randomized to each arm. Of those who completed the intervention, 30 (97%) participants reported that they were satisfied/very satisfied with the intervention. Twenty-seven (87%) participants indicated that they would recommend the program to others, and 26 (84%) thought that the program was worth their time. Intervention participants demonstrated several positive nonsignificant improvements versus the control group at six months: burden (P= 0.53), depressive symptoms (P= 0.19), general self-efficacy (P= 0.14), QoL (P= 0.17) and fatigue (P= 0.15).

Conclusions

Study findings demonstrate that combined M-CBT and telephone counseling intervention appear to be feasible, but did not lead to nonsignificant improvements in outcome measures such as burden in family carers of persons with AMD.

## Introduction

Age-related macular degeneration (AMD) is a leading cause of irreversible visual impairment in adults aged 50 or older in the developed world [1]. In 2020, approximately 196 million individuals worldwide were estimated to have AMD, and by 2040, this number is expected to increase to 288 million due to the projected increase in the aging population [1]. AMD is a chronic, degenerative retinal condition that leads to central vision loss, reduced functional independence, and poorer quality of life (QoL) [2,3]. As such, they often require regular physical and emotional support beyond what is provided by the healthcare system [4]. Family members of relatives with AMD are often called on to fulfill this role despite not receiving adequate support and training, which can be a burdensome task [5]. Our previous research shows that carers of people with AMD endure significant physical burden, emotional distress, increased financial stress, and disruptions due to their lifestyle and retirement plans as a direct consequence of the AMD caregiving experience [6-8].

Despite the importance of providing information and support to assist family caregivers, there are currently no interventions targeted toward family caregivers of AMD patients. Services tailored to the needs of family caregivers can improve their problem-solving skills, reduce distress, and enhance physical and mental well-being [9]. Cognitive behavioral therapy (CBT) is a form of psychotherapy that has been shown to decrease the burden and distress in caregivers of patients with chronic conditions [10-12]. CBT targets and challenges maladaptive thoughts to foster the development of long-lasting cognitive and behavioral skills that can be used to overcome difficult or stressful situations encountered in the line of care [13,14].

The novel intervention in this study was the first program globally to provide a tailored support service for family caregivers of individuals with AMD. The intervention comprised mail-delivered CBT (M-CBT), telephone-delivered group counseling, and education on available support. M-CBT involving written materials that were posted to caregivers was chosen over standard face-to-face CBT to enable them to review the material as often as needed. There is evidence from randomized controlled trials (RCTs) for the effectiveness of M-CBT in a diverse range of conditions [15,16]. This multicenter RCT aimed to investigate the feasibility and efficacy of this novel multimodal intervention for family carers of people with AMD when compared with usual care only. It was hypothesized that caregivers with the intervention would experience significantly reduced perceived caregiver burden, depression, fatigue, and improved health-related QoL and self-efficacy scores.

This article was previously posted to the Qeios preprint server on May 9, 2023 (https://doi.org/10.32388/8JYANC.2).

## Materials and methods

Study design and participants

This was a multicenter, two-arm RCT with an intervention group and a wait-list control group. The study protocol has been published previously [17]. Ninety-four family carers of individuals with AMD were recruited from private ophthalmology clinics in Sydney, Australia and the Macular Disease Foundation Australia (MDFA) client database between January 2017 and May 2020. Participants were eligible if they were (1) aged 18 years or older; (2) family caregiver to an individual diagnosed with AMD and related to the care recipient (e.g., spouse, child, or sibling); and (3) provided written informed consent to engage in a 10-week therapeutic intervention over a three-month period. Participants were excluded if they were unable to speak and understand English.

Randomization and blinding

Participants were randomly assigned to the intervention or control group using a randomization sequence generated centrally using permuted blocks of the mixed size that were stratified by the recruitment site to ensure equal participant numbers. Assignments to the intervention or control group were managed centrally by an individual who was not part of the recruiting or treating team to ensure allocation concealment. Forty-seven participants were assigned to the intervention group, and 47 participants were assigned to the control group. It was not possible to blind the investigators or participants due to the nature of the intervention.

Trial procedures

The intervention group received a 10-week multimodal support service program consisting of five M-CBT modules and five Talk Link group counseling sessions that were delivered weekly on an alternating basis. The M-CBT modules included education and/or information on AMD; stress response; healthy lifestyle habits; challenging negative thoughts and cognitive distortions; and problem-solving skills. Participants were telephoned following each M-CBT module for a brief discussion to address any queries and encourage the application of skills from the modules. The Talk Link group counseling sessions were one-hour sessions conducted over the phone and included groups of six to eight caregivers and two trained facilitators. These sessions were aimed at identifying issues relating to the caregiving experience and reinforcing CBT skills taught in the previous week.

Participants in the control group received reading materials about AMD and caring for persons with the condition, with the opportunity to receive the intervention at the end of the study period [17].

Outcomes

A preintervention baseline questionnaire was administered to all family caregivers to obtain demographic information including age, sex, employment status, their own health status, living arrangements, marital status, whether they are the sole caregiver, relationship to the care recipient, and years of education. The baseline questionnaire was readministered to carer participants at six months post-intervention to capture changes in (i) Caregiver Burden Scale (CBS), assessing caregiver burden; (ii) Centre for Epidemiologic Studies Depression Scale-10 (CESD-10), screening for the presence of depressive symptoms; (iii) General Self-Efficacy Scale (GSE), assessing one’s beliefs in their ability to succeed in specific situations; (iv) Fatigue Severity Scale (FSS), determining the impact of fatigue on an individual’s ability to carry out activities and physical functioning; and (v) EQ-5D-5L and the visual analogue scale (EQ-VAS), measuring health-related QoL. Treatment acceptability was assessed by having participants complete a feedback form including five-point Likert scales to report satisfaction and adherence with the intervention.

Changes in outcome measures between baseline and six months were determined using paired t-tests for continuous variables (self-efficacy and QoL) and McNemar’s test for binary “Yes/No” variables (CESD-10, FSS, and CBS). The primary outcome was the change in subjective caregiver burden measured using the CBS. Secondary outcomes included change from baseline in fatigue, depressive symptoms, health-related QoL (EQ-5D-5L), self-efficacy scores, and treatment acceptability and feasibility.

Ethics approval

Ethics approval was obtained from The University of Sydney Human Research Ethics Committee (approval number 2016/793). This study was performed in accordance with the tenets of the Declaration of Helsinki. The trial registration number is ACTRN12616001461482.

## Results

**Figure 1:**
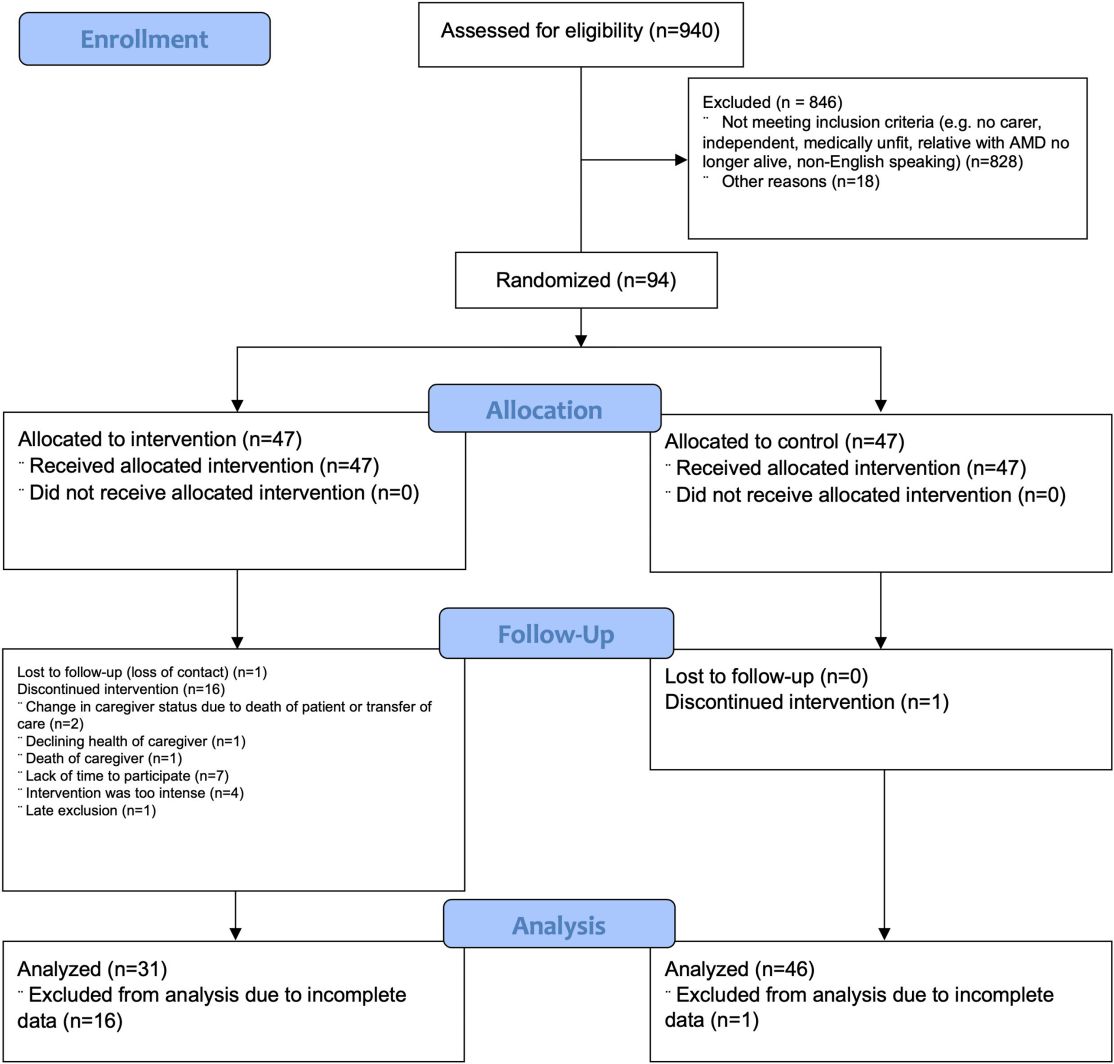
CONSORT trial flow diagram showing participant flow throughout each process of the RCT CONSORT, Consolidated Standards of Reporting Trials; RCT, randomized controlled trial

Between January 2017 and May 2020, a total of 940 patients were approached, and 94 were recruited and randomized (Figure 1). At the conclusion of the study, 17 participants withdrew from the study (one control and 16 intervention) due to the death of the caregiver (n = 1), declining health of the caregiver (n = 1), lack of time to participate (n = 7), change in caregiver status due to the death of an AMD patient (n = 2), intervention being too intense (n = 4), loss of contact (n = 1), and late exclusion (n = 1). There were no significant differences in baseline characteristics between control and intervention participants (Table 1).

**Table 1:** Study characteristics of family carers and patients at baseline NEI VFQ-25, National Eye Institute Visual Function Questionnaire 25

	Intervention	Control	P-value
Carer variables			
Age (mean years ± SD)	64.5 ± 13.3	62.5 ± 14.3	0.49
Female sex, n (%)	35 (74.5)	38 (80.9)	0.46
Dependency on carer – High level of dependency, n (%)	19 (40.4)	20 (42.6)	0.9
General health status – Substantial comorbidity, n (%)	16 (34.0)	11 (23.4)	0.25
Patient variables			
Age (mean years ± SD)	82.9 ± 9.6	83.4 ± 9.7	0.79
Female sex, n (%)	31 (66.0)	31 (66.0)	1
General health status – Substantial comorbidity, n (%)	23 (48.9)	24 (51.1)	0.84
NEI VFQ-25 score (mean ± SD)	55.5 ± 25.3	50.4 ± 26.7	0.34

Table 2 shows the differences in outcome measures between the control and intervention groups at six-month follow-up. Carers in the intervention versus the control arm at six-month follow-up had non-statistically significant reductions in caregiver burden scores (mean difference (95% CI), -2.70 (-11.10, 5.70); P = 0.53) and fewer carers with significant depressive symptoms (RR, 0.61 (95% CI: 0.32, 1.13); P = 0.19) and fatigue (RR, 0.56 (95% CI: 0.25, 1.25); P = 0.15). The intervention arm also showed higher but nonsignificant self-efficacy (mean difference (95% CI), 1.86 (-0.53, 4.25); P = 0.14) and QoL VAS scores (mean difference (95% CI), 5.45 (-2.26, 13.16); P = 0.17) compared to the control arm.

**Table 2:** Comparison between study arms: baseline versus six months post-intervention Values are mean ± SD or n (%). ^#^ Reported as RR (95% CI) CESD-10, Centre for Epidemiologic Studies Depression Scale-10; EQ-5D, European Quality of Life 5 Dimensions; FSS, Fatigue Severity Scale; QoL, quality of life; VAS, Visual Analogue Score

	Baseline			Six months post-intervention			
Measure	Control	Intervention	P-value	Control	Intervention	Mean difference (95% CI)	P-value
Carer burden score	27.96 ± 18.81	24.79 ± 16.61	0.39	28.70 ± 17.97	26.00 ± 18.41	-2.70 (-11.10, 5.70)	0.53
Self-efficacy score	32.55 ± 4.66	32.36 ± 5.09	0.85	30.80 ± 5.11	32.66 ± 5.25	1.86 (-0.53, 4.25)	0.14
QoL: EQ-5D score	0.81 ± 0.19	0.79 ± 0.15	0.55	0.77 ± 0.16	0.80 ± 0.19	0.03 (-0.05, 0.11)	0.54
QoL: VAS score	76.45 ± 19.40	77.74 ± 14.41	0.71	73.83 ± 16.69	79.28 ± 16.63	5.45 (-2.26, 13.16)	0.17
Problematic fatigue (FSS ≥4)^#^	16 (34.0)	12 (38.3)	0.67	22 (47.8)	9 (31.0)	0.61 (0.32, 1.13)	0.15
Significant depressive symptoms (CESD-10 ≥10)^#^	11 (23.4)	8 (25.5)	0.81	16 (34.8)	6 (20.7)	0.56 (0.25, 1.25)	0.19

Of those who completed the intervention, 30 (97%) participants reported that they were satisfied/very satisfied with the intervention. Of those who participated in the telephone counseling component, 14 (93%) were satisfied or very satisfied. Twenty-one (68%) participants self-reported high adherence to the intervention. Twenty-seven (87%) participants also indicated that they would recommend the program to others, and 26 (84%) participants thought that the program was worth their time.

## Discussion

The provision of consistent education and support to alleviate the burden and distress of caregivers of AMD patients remains a challenge. This was the first study to evaluate an intervention targeting the carer burden among family carers of people with AMD. In this study, we implemented a multimodal program delivered remotely over 10 weeks for caregivers of AMD patients across several private ophthalmology clinics and the MDFA database. The study found no statistically significant effect on key outcome measures at six months post-intervention.

Although the results were not statistically significant, it was not possible to rule out the presence of clinically significant benefits in the multimodal intervention [18]. Statistical significance alone does not necessarily constitute clinically significant improvements or meaningful change when interpreting a study’s outcome for application to patient care [19,20]. Clinically meaningful changes refer to those that improve an individual’s QoL, social function, as well as physical and mental well-being [21]. The results showed promising nonsignificant differences in several secondary outcome measures, including reduction in depression, fatigue, and improved QoL between the intervention and control group in the expected direction.

In our study, the VAS QoL score, which measured overall general well-being, was approximately six units higher in the intervention versus control group after six months [22]. It has been argued that any change in QoL itself may be considered clinically significant [23]. While the CI contains zero, it lies largely in the positive direction and covers clinically meaningful beneficial values. This may be interpreted as clinically significant and potentially beneficial, but further studies with larger sample sizes are needed to clarify this.

There was a 39% reduction (RR 0.61 (95% CI, 0.32-1.13)) in significant depressive symptoms and a 44% reduction (RR 0.56 (95% CI, 0.25-1.25)) in fatigue between the intervention and control arms, with the CIs lying largely in the clinically meaningful direction. A review conducted on caregiver intervention research in dementia showed that changes in depressive symptoms from as little as 0.75% to 10.5% can be clinically meaningful [24]. CBT is highly effective in treating depression, anxiety, and fatigue [25]. However, the effectiveness of CBT in individual studies involving caregivers has been difficult to evaluate due to the low participant numbers in these studies. A systematic review and meta-analysis of seven RCTs assessing the effect of CBT on depression in caregivers of dementia patients found that each included study resulted in non-statistically significant but clinically meaningful outcomes [2]. Each study had a caregiver sample size ranging from 13 to 68 caregivers. However, when the studies were pooled to create a sample size of 161 caregivers, there was a large statistically significant decrease in the presence of significant depressive symptoms as indicated by the CESD-10 score in the CBT intervention compared to control groups [2]. Hence, the low sample size in our study most likely led to type II errors, which resulted in non-statistically significant results. There was also a relatively high withdrawal rate from the intervention group. However, this was comparable to other studies examining the efficacy of CBT for family carers of relatives with dementia, with one such study reporting 17% of participants failing to complete the CBT intervention sessions [26]. Another study involving a CBT-based problem-solving intervention for family carers of stroke victims reported that 30.9% of participants did not complete the intervention, citing similar reasons to those observed in our study [27].

Future studies investigating combined interventions should consider using more recruitment and participant retention strategies. Recruitment strategies could include the use of more recruitment centers, including private and public ophthalmology clinics, retinal disease, and low vision support services, as well as the use of public service announcements via community presentations, targeted newsletters, and brochures with a detailed description of the intervention in plain language. Participant retention strategies include financial incentives, more options for alternative data collection methods (e.g., face-to-face, via telephone), and personal reminders. Furthermore, inclusion criteria could be made less specific to include informal caregivers who were not direct relatives of the patient.

Apart from increasing the sample size, the number of CBT sessions could have been increased to improve the effect size. There is evidence stating that remote delivery of CBT required longer interventions to achieve favorable treatment effects when compared to face-to-face delivery [28]. Literature has shown that around 10-12 CBT sessions are required to demonstrate sustainable improvements in psychological well-being [29]. Future trials involving remote CBT should aim to increase the number of CBT sessions.

The intervention was feasible, as shown by the relatively high satisfaction and adherence rate, as well as the lack of adverse effects. There are currently no other treatments that exist to address the burden experienced by caregivers of AMD patients. The results of this trial are inconclusive because a clinically meaningful treatment effect cannot be ruled out. If proven to be effective through larger studies, the multimodal intervention, including CBT and group counseling, can be a cost-effective method of reducing caregiver burden.

A key study strength is that this was the first evaluation of a novel multimodal intervention addressing the burden on caregivers of AMD patients based on a rigorous experimental design. This multidisciplinary intervention was developed in collaboration with clinical psychologists, ophthalmologists, and peak advocacy groups representing individuals with AMD and family carers. Our methodological approach had several limitations. Firstly, the pre-planned sample size was not achieved. The target sample size was 194 caregivers who would have provided 80% power to detect a statistically significant effect size of 0.5 standard deviations in the primary outcome of caregiver burden; however, the realized sample size was 94 caregivers. Combined with the large number of withdrawals in the intervention arm due to non-trial-related reasons, this resulted in the study having a low statistical power, and hence non-statistically significant results due to type II error were likely. Reasons for the low sample size were restricted recruitment time due to COVID-19, the low number of recruitment centers, recruitment in low health literacy suburbs, and strict inclusion criteria (e.g., caregivers had to be related to the patient). The relatively lower follow-up rate in the intervention group could also be because of the high mean age of caregivers of AMD patients, who often need to concurrently manage their own health. Recruitment of younger caregivers may help improve compliance and reduce loss to follow-up. Secondly, due to the nature of the intervention, blinding of participants and staff involved in the project was not possible. This could have led to response bias when participants were answering their questionnaires. Finally, the study did not evaluate the long-term effects of the intervention in the past six months. Future trials should aim to assess long-term effects after six months of intervention.

## Conclusions

This study implemented a remotely delivered multimodal intervention to provide support to family carers of people with AMD across several ophthalmology practices and the MDFA database. The intervention was not associated with statistically significant improvements in caregiver burden among caregivers of AMD patients. However, the intervention was feasible and showed promising but not statistically significant results with regard to reducing depression and fatigue and improving overall general well-being. Whether remotely delivered multimodal programs offer a means of relieving the burden and improving the well-being of caregivers of AMD patients remains uncertain. The results of our study will provide further insight to guide the future development of interventions for caregivers of AMD patients. The study protocol and intervention appear to be feasible; however, the recruitment procedure will have to be improved. Considering the COVID-19 pandemic, remotely delivered formats such as M-CBT and telephone counseling could be especially useful for allowing the continued provision of care and therefore, require further larger studies to evaluate the efficacy.
